# Medical Attendance for Lower Urinary Tract Symptoms Is Associated with Subsequent Increased Risk of Outpatient Visits and Hospitalizations Based on a Nationwide Population-Based Database

**DOI:** 10.1371/journal.pone.0057825

**Published:** 2013-03-05

**Authors:** Ming-Ping Wu, Shih-Feng Weng, Ya-Wen Hsu, Jhi-Joung Wang, Hann-Chorng Kuo

**Affiliations:** 1 Division of Urogynecology, Department of Obstetrics and Gynecology, Chi Mei Foundation Hospital, Tainan, Taiwan Authority; 2 Center of General Education, Chia Nan University of Pharmacy and Science, Tainan, Taiwan Authority; 3 Department of Hospital and Health Care Administration, Chia Nan University of Pharmacy and Science, Tainan, Taiwan Authority; 4 Department of Medical Research, Chi Mei Medical Center, Tainan, Taiwan Authority; 5 Department of Urology, Buddhist Tzu Chi General Hospital and Tzu Chi University, Hualien, Taiwan Authority; College of Pharmacy, University of Florida, United States of America

## Abstract

**Introduction:**

Lower urinary tract symptoms (LUTS), which encompass storage, voiding, and postmicturition symptoms, are highly prevalent and recognized globally. Based on a nationwide population-based database, this study tests the hypothesis that medical attendance for LUTS is associated with a subsequent increase in the number of outpatient visits and hospitalizations, with differences among medical specialties and age groups.

**Methods:**

Participants were selected from a random population sample of approximately one million people as a representative cohort of National Health Insurance (NHI) enrollees in Taiwan. Participants had at least three outpatient service claims with a coding of LUTS during the recruitment period 2001–2004. Both the LUTS group and non-LUTS control group were monitored for subsequent outpatient visits and hospitalizations, excluding LUTS-related healthcare services, for 2 years following the index date. The results were categorized based on medical specialty and age group.

**Results:**

The outpatient visit rates (no. per person-year) and adjusted incidence rate ratios (IRRs) (95% confidence interval (CI) were significantly higher in urology (4.51, 95%CI 4.15–4.91) and gynecology (1.82, 95%CI 1.76–1.89) for the LUTS group. They were also significantly high in other departments, including internal medicine (1.25), general practice (1.13), Chinese medicine (1.77), family medicine (1.19), surgery (1.38), and psychiatry (1.98). Similarly, the hospitalization rate (no. per 1000 person-year) and adjusted IRRs (95% CI) were significantly higher in urology (5.50, 95% CI = 4.60–6.50) and gynecology (1.60, 95% CI = 1.35–1.90), as well as in internal medicine (1.55) and surgery (1.56), but not in psychiatry (1.12). Furthermore, the IRRs differed among 3 age groups.

**Conclusions:**

A significantly higher number of outpatient visits and hospitalizations were observed for individuals with LUTS, compared to the control group, and the effects differed with the advancement of age. This study broadens understanding of LUTS by viewing their impact on healthcare services with multiple and overlapping systems, rather than considering them exclusively as symptoms of traditional diseases of the bladder and urethra.

## Introduction

Lower urinary tract symptoms (LUTS) is an umbrella term that encompasses all urinary symptoms, including storage, voiding, and postmicturition symptoms [1]. LUTS have gained global attention because of their high prevalence and impact on a person’s quality-of-life (QoL). The prevalence of LUTS increases with age among the general population [2]–[4]. LUTS progress with only a minority of cases that show regression [5]. According to the EPIC epidemiology study conducted in five European countries using definitions established by the 2002 International Continence Society (ICS), the existence of LUTS was as high as 64.3%, with a total of 19,165 people having at least one of the symptoms. Nocturia was the most common of the LUTS, (48.6% in men and 54.5% in women). Storage, voiding, and postmicturition symptoms was 51.3%, 25.7%, and 16.9% in men and 59.2%, 19.5%, and 14.2% in women, respectively. Overactive bladder (OAB) was 11.8%. Rates were similar between men and women and increased with the advancement of age. Furthermore, LUTS increase worldwide burden over time [6]. By 2018, an estimated 2.3 billion people will be affected by at least one of the LUTS (an 18.4% increase), 546 million by OAB (20.1%), 423 million by urinary incontinence (UI) (21.6%), and 1.1 billion by LUTS/bladder outlet obstruction (BOO) (18.5%). The regional burden of these conditions is estimated to be greatest in Asia [6]. In Taiwan, a total of 53.7% of the women sampled suffered from UI and related symptoms [2].

LUTS increase with the advancement of age among the general population [2]–[4]. In a recent study, we found that demand for healthcare service, with separate subtype distributions, also increased with the advancement of age [7]. Aging is a significant factor in the prevalence of LUTS, and the problems associated with aging is a worldwide phenomenon [8], [9]. In Taiwan, the problem of aging is accelerated and severe. The older population (≥65 years) was 7.10% of the total population in 1993, 8.62% in 2000, 10.00% in 2006, and 10.63% in 2009. The aging index (older population ≥65 years/youth population <15 years) was 26.41% in 1993, 40.85% in 2000, 55.17% in 2006, and 65.05% in 2009 (Department of Statistics, Ministry of Interior, Executive Yuan, Taiwan). The pathogenesis of LUTS is not completely understood, however it is considered a multifactorial process (including neurologic, vascular, and connective tissue disorders) [10]. Furthermore, LUTS are known to have a negative impact on health-related quality of life (HRQL) [5], [11]. OAB, with and without incontinence, has a clinically significant impact on QoL, quality-of-sleep, and mental health in both men and women [12]. A significant age-related increase in the International Prostate Symptom Score (IPSS) and QoL scores was observed in both genders [13]. LUTS may also cause a decrease in work productivity, activity impairment, and work absenteeism [11].

When encountering LUTS, patients may either initiate a consultation with their health-care provider, or alter their daily activities (e.g., limiting fluid intake, using absorbent products, doing physiotherapy or exercising) [2], [14]. Moreover, approximately two-thirds of incontinent women restrict their social activities (due to fear of a lack of toilet facilities in the event of wetting or leakage), and approximately 19% of incontinent women experience an altered sex life [2]. One study reported that LUTS/OAB increase health risks [15], however, whether LUTS result in an increased number of outpatient visits and hospitalizations has not yet been investigated based on a population-based registry. Furthermore, whether the effects of LUTS on healthcare services differ among medical specialties and age groups is still undetermined. Based on a nationwide population-based database among National Health Insurance (NHI) enrollees in Taiwan, this study tests the hypothesis that medical attendance for LUTS is associated with a subsequent increase in the number of outpatient visits and hospitalizations, with differences among medical specialties and age groups. Furthermore, this study examines whether LUTS are a precursor to the development of other medical or surgical conditions and, therefore, whether they have an impact on health care services that involve broader clinical practices and even public policy.

## Methods

### Data Source

Data for this study were obtained from a random sample of approximately one million enrollees (approximately 5% of Taiwan’s population) as a representative cohort from the National Health Insurance Research Database (NHIRD) in Taiwan, which covers all outpatient and inpatient medical benefit claims. No statistically significant differences in age, gender, or costs between the sample group and all enrollees in Taiwan exist. Details of the NHIRD are described in our previous studies [7], [16]. Briefly, NHIRD was established by the National Health Research Institute to promote research in existing and emerging medical issues in Taiwan. The NHI program was implemented in March 1995 to offer a comprehensive, unified, and universal health insurance program to all citizens. Therefore, a fair share of risk-pooling for NHI should be expected. All citizens who have established a registered domicile for at least 4 months in Taiwan are eligible for NHI enrollment. The Bureau of NHI (BNHI) has contracted with most medical institutions in Taiwan. As many as 93.1% of people in Taiwan has joined the NHI program since 1996, with a coverage increasing up to 99.2% in 2009. The NHIRD provides a patient’s encrypted identification number, gender, date of birth, dates of outpatient visits, as well as the International Classification of Diseases, 9th Revision, Clinical Modification (ICD-9–CM) codes of diagnoses and procedures, and details of prescriptions and expenditure amounts. All NHI datasets can be interlinked with each individual personal identification number.

### The Definition of LUTS

We identified individuals for the study group (LUTS group) as those who had at least three out-patient service claims during 2001–2004 with the following coded conditions: storage symptoms, including hypertonicity of bladder (ICD-9 CM code 596.51), frequency and polyuria (788.4), stress urinary incontinence in female (625.6) and male (788.32), urge incontinence (788.31), nocturnal enuresis (788.36), nocturia (788.43), mixed incontinence (788.33); voiding symptoms, including retention of urine (788.2), splitting & slowing of urine stream (788.6), and post-void dribbling (788.35). Males with benign prostatic hyperplasia (enlargement) (600) were also identified and the symptom categorized as an associated symptom, whereas LUTS/BPH without storage and voiding symptoms were categorized as unclassified male LUTS. The American Urologic Association (AUA) 2010 guidelines for BPH defines LUTS/BPH as LUTS secondary to BPH; therefore, LUTS is inseparable from clinical BPH [17].

### Study Participants and Comparison Group

Participants in the study (LUTS group, n = 39901) were men and women who had at least three outpatient service claims at hospitals of different accreditation levels or at local medical clinics with coded LUTS during the recruitment period 2001–2004. To reduce and avoid wandering comparison of risk and selection biases such as Berkson’s bias (e.g., selecting the study group from a hospital population, and the control group from among the hospitalized population) [18], we selected both study and comparison groups from the random sample of one million cohort with outpatient bases. Controls (those in the non-LUTS group matched one-to-one for each LUTS patient, n = 39901) were individuals not diagnosed with LUTS and randomly selected from the dataset. They were matched by sex, age (±30 days), and index date. The index date for participants with LUTS was the date of their first registration. The year of the index-date of each participant with LUTS was used to create the index date for each control subject. Comorbid hypertension (HTN) and diabetes (DM) in these 2 cohorts were also matched because these two chronic diseases are most commonly associated with frequency of the health-care seeking. All recruited cases were monitored for outpatient visits and hospitalizations for 2 years following the index date, except those who expired during the follow-up period. Subsequent outpatient visits and hospitalizations of the LUTS and non-LUTS groups were identified, excluding LUTS-related health-care services. Subsequent health-care services, including all-cause and specialty-specific hospitalizations, were classified according to medical specialty and age group (<40, 40–60, ≥60 years of age). The classification of medical specialties and their subspecialties was based on codes established by the National Health Research Institute. Internal Medicine includes the subspecialties of gastroenterology, cardiology, nephrology, rheumatology, medical oncology, endocrinology, infectious diseases, and chest medicine. Surgery includes colorectal, cardiovascular, thoracic, and gastrointestinal surgery. The subspecialties were categorized under either internal medicine or surgery, unless otherwise specialized. For example, neurology, orthopedic surgery, and neurosurgery were identified as separate departments. For privacy protection, the unique identifiers of the patients and institutes were scrambled cryptographically to ensure anonymity. Confidentiality was assured by abiding by data regulations of the Bureau of NHI, and institutional review board approval was waived.

### Measures and Statistical Analysis

Demographical information, including age, sex, race, insurance amount, and region, were obtained from the BNHI-insured's file. Age was grouped into three categories: 18–39, 40–59, and 60 or more years of age. The insurance amount was classified into one of three categories: less than US$640 (NTD20,000), US$640–US$1280 (NTD 20,000–39,999), and US$1281 (NTD 40,000) or more. In terms of geographic distribution, participants were classified into one of four regions: northern, central, southern, and eastern.

Descriptive statistical analyses using t-test for continuous variables and Chi-square test for categorical variables were conducted to compare differences between the LUTS group and the control group in terms of socio-demographic characteristics and comorbidities. The incidence rate was calculated as the number of outpatient visits or hospitalizations during the follow-up period, divided by the total person-years for each group for both LUTS and non-LUTS groups. The risk of outpatient visits or hospitalization between the LUTS group and the non-LUTS group were compared by estimating the incidence rate ratio (IRR) using Poisson regression. Prior to implementing the models, we assessed possible overdispersion of the count outcome data by testing whether the negative binomial dispersion parameter was significantly different from zero. Because it was significant, a Poisson regression model using SAS PROC GENMOD taking overdispersion into account was used. The adjusted incidence rate ratio was calculated by multivariate Poisson regression to compare the incidence rate between LUTs and non-LUTS groups after adjusting for possible confounders such as age, gender, income, area, HTN, DM, hyperlipidemia, and coronary arterial disease (CAD). Other potential confounders such as education, marital status, alcohol use, tobacco use, and measures of baseline health status (e.g., obesity and parity) were not available in the dataset. Individual medical specialties were derived from the data and defined in tables. Subspecialties were categorized under internal medicine or surgery, unless otherwise specialized (e.g., neurology and neurosurgery were identified as separate departments). All analyses were performed using SAS software version 9.3 (SAS Institute, Cary, NC, USA). For the descriptive statistical analysis, a p-value of less than 0.05 was considered significant. However, because of the large number of hypothesis tests performed (multiple testing issue), a more precise p-value of 0.0029 (0.05/17) with Bonferroni correction (significance level/no. of specialty areas) was considered significant.

## Results

The participants were matched for age, gender, HTN, DM in the LUTS group and the control group. Demographic information for LUTS and non-LUTS individuals is shown in Table 1. Although several LUTS-related confounding factors (e.g., body mass index, parity, alcohol use, and tobacco use) were significant to the study, information for these factors was not available due to the characteristics of the registry claim database. Outpatient visits and hospitalizations of the LUTS and control groups were identified, with the exclusion of LUTS-related healthcare services. As expected, the rate of outpatient visits was higher in gynecology (3.00 for the LUTS group vs. 0.43 for the control group) (no. per one person-year) and urology (0.43 vs. 0.09), with an adjusted IRR of 1.82 (95% confidence interval (CI) = 1.76–1.89) and 4.51 (95% CI = 4.15–4.91), respectively. The total outpatient visit rate (except for gynecology and urology) (no. per one person-year) for the LUTS group and non-LUTS group was 24.53 and 18.71, respectively. After adjusting for age, gender, HTN, DM, hyperlipidemia, and CAD, the adjusted IRR was 1.31 (95% CI = 1.29–1.32). In addition to the IRRs for gynecology and urology, those for other departments were significantly higher as well (internal medicine (1.25, 95% CI = 1.23–1.27); general practice (GP) (1.13, 95% CI = 1.10–1.13); Chinese medicine (1.77, 95% CI = 1.71–1.83); family medicine (1.19, 95% CI = 1.15–1.22); surgery (1.38, 95% CI = 1.33–1.44); psychiatry (1.98, 95% CI = 1.80–2.17); and emergency room (ER) (1.56, 95% CI = 1.47–1.65)). All *p*-values were <0.001. Data are shown in detail in Table 2.

**Table 1:** Demographic information for LUTS and non-LUTS individuals.

	LUTS(N = 39901)(%)	Non-LUTS(N = 39901)(%)	*P* value*
Age	55.35±16.94	55.33±16.92	0.906
Gender Female	16178 (40.55)	16178 (40.55)	1.000
Male	23723 (59.45)	23723 (59.45)	
AreaNorthern	18273 (45.80)	19424 (48.68)	<0.001
Central	7668 (19.22)	7080 (17.74)	
Southern	21987 (32.55)	12372 (31.01)	
Eastern	973 (2.44)	1025 (2.57)	
Income <20,000	30351 (76.07)	30221 (75.74)	0.062
20,000∼40,000	5850 (14.66)	6072 (15.22)	
>40,000	3700 (9.27)	3608 (9.04)	
Comorbidity			
HTN Yes	9689 (24.28)	9689 (24.28)	1.000
No	30212 (75.72)	30212 (75.72)	
DM Yes	4234 (10.61)	4234 (10.61)	1.000
No	35667 (89.39)	35667 (89.39)	
Hyperlipidemia Yes	2524 (6.33)	2248 (5.63)	<0.001
No	37377 (93.67)	37653 (94.37)	
CAD Yes	3298 (8.27)	2772 (6.95)	<0.001
No	36603 (91.73)	37129 (93.05)	

* *p*-value is from the *t*-test for continuous variables and Chi-square test for categorical variables;

Individuals were matched by age, gender, HTN, and DM;

LUTS: lower urinary tract symptom; HTN: hypertension; DM: diabetes mellitus; CAD: coronary arterial disease.

**Table 2:** Incidence rate ratio (IRR) of outpatient visits among LUTS and non-LUTS individuals.

	LUTS(N = 39901)		Non-LUTS(N = 39901)		Unadjusted		Adjusted	
Department	Visits	Rate*	Visits	Rate*	IRR** (95%CI)	*P* value	IRR***(95%CI)	*P* value
Gynecology#	96063	3.00	52810	1.64	1.83 (1.76–1.90)	<0.001	1.82 (1.76–1.89)	<0.001
Urology	33345	0.43	7417	0.09	4.52 (4.14–4.93)	<0.001	4.51 (4.15–4.91)	<0.001
Internal Medicine	441497	5.64	354377	4.51	1.25 (1.22–1.28)	<0.001	1.25 (1.23–1.27)	<0.001
GP	310219	3.96	266766	3.39	1.13 (1.10–1.16)	<0.001	1.13 (1.10–1.16)	<0.001
Chinese Medicine	220970	2.82	123436	1.57	1.80 (1.74–1.86)	<0.001	1.77 (1.71–1.83)	<0.001
Family Medicine	198575	2.54	167058	2.13	1.19 (1.15–1.24)	<0.001	1.19 (1.15–1.22)	<0.001
Ophthalmology	108055	1.38	83179	1.06	1.30 (1.26–1.35)	<0.001	1.30 (1.27–1.34)	<0.001
ENT	108113	1.38	77275	0.98	1.41 (1.36–1.45)	<0.001	1.41 (1.36–1.46)	<0.001
Surgery	77012	0.98	56024	0.71	1.38 (1.32–1.44)	<0.001	1.38 (1.33–1.44)	<0.001
Psychiatry	34573	0.44	17468	0.22	1.99 (1.81–2.18)	<0.001	1.98 (1.80–2.17)	<0.001
ER	16461	0.21	10731	0.14	1.54 (1.45–1.64)	<0.001	1.56 (1.47–1.65)	<0.001
Other	414388	5.30	314173	4.00	1.32 (1.30–1.35)	<0.001	1.32 (1.30–1.35)	<0.001
**Total**	**1919470**	24.53	**1470487**	18.71	**1.31 (1.30–1.33)**	**<0.001**	**1.31 (1.29–1.32)**	**<0.001**

* per one person-year. The total person-year was 78259.1 in the LUTS group, and 78606.5 in the non-LUTS group.

** IRR: incidence rate ratio for the LUTS group vs. the non-LUTS group;

*** adjusted by age, gender, income, area, HTN, DM, hyperlipidemia, and CAD;

# only women were included in gynecology. GP: general practice; ENT: ear, nose and throat; ER: emergent room;

Among the 3 age groups in both the LUTS and non-LUTS groups, the rate of outpatient visits increased with the advancement of age in urology, internal medicine, general practice, family medicine, ophthalmology, and surgery, whereas the rates decreased in gynecology, Chinese medicine, ENT, and psychiatry. The overall IRRs of outpatient visits, excluding gynecology and urology, were the highest in the younger group (1.47, 95% CI = 1.43–1.51), followed by the middle-aged (40 to 60 years) group (1.35, 95% CI = 1.33–1.38), and the older group (over 60 years of age) (1.25, 95% CI = 1.23–1.51), (i.e., the IRRs decreased in the older group (Table 3)). The IRRs of outpatient visits decreased with the advancement of age in internal medicine, general practice, Chinese medicine, ophthalmology, and psychiatry. On the contrary, the IRRs increased with the advancement of age for gynecology (Table 3). With the inclusion of LUTS-related healthcare services, outpatient visit rates to urology increased among men and women, but outpatient visits to gynecology decreased among women with the advancement of age in both the LUTS and non-LUTS groups, although both adjusted IRRs increased. The adjusted IRRs were 1.88, 2.13, and 2.74 in gynecology, and 11.4, 14.3, and 20.7 in urology among the three age groups (data not shown).

**Table 3:** Incidence rate ratio (IRR) of outpatient visits among LUTS and non-LUTS individuals categorized by age group.

Grouping	Age < 40					40 ≦Age <60					Age <$>\raster="rg1"<$>60				
	LUTS(N = 8099)		non-LUTS(N = 8108)		AdjustedIRR**(95%CI)	LUTS(N = 14552)		Non-LUTS(N = 14538)		AdjustedIRR**(95%CI)	LUTS(N = 17250)		Non-LUTS(N = 17255)		AdjustedIRR**(95% CI)
Department	Visits	Rate*	Visits	Rate*		Visits	Rate*	Visits	Rate*		Visits	Rate*	Visits	Rate*	
Gynecology#	47011	4.32	26882	2.46	1.75†	38456	2.99	20955	1.63	1.83†	10596	1.28	4973	0.59	2.16†
					(1.65–1.84)					(1.73–1.93)					(1.93–2.43)
Urology	5163	0.32	1074	0.07	4.89†	12036	0.42	2828	0.10	4.27†	16146	0.49	3515	0.11	4.61†
					(4.16–5.75)					(3.75–4.85)					(4.01–5.30)
Internal Medicine	30932	1.91	20006	1.23	1.53†	135020	4.67	100827	3.48	1.33†	275513	8.31	233544	6.98	1.19†
					(1.44–1.63)					(1.29–1.38)					(1.16–1.22)
GP	34753	2.15	27468	1.69	1.25†	96378	3.33	84938	2.93	1.13†	168695	5.09	154360	4.61	1.10†
					(1.18–1.33)					(1.09–1.19)					(1.07–1.15)
Chinese Medicine	59515	3.68	29732	1.83	1.96†	94055	3.25	49886	1.72	1.86†	67400	2.03	43818	1.31	1.54†
					(1.84–2.08)					(1.77–1.96)					(1.45–1.64)
Family Medicine	19633	1.21	13882	0.86	1.38†	59920	2.07	51026	1.76	1.17†	119022	3.59	102150	3.05	1.17†
					(1.28–1.49)					(1.11–1.23)					(1.12–1.22)
Ophthalmology	11013	0.68	7581	0.47	1.45†	27921	0.97	19442	0.67	1.43†	69121	2.08	56156	1.68	1.24†
					(1.36–1.56)					(1.36–1.52)					(1.19–1.29)
ENT	28435	1.76	21739	1.34	1.32†	45271	1.57	30926	1.07	1.46†	34407	1.04	24610	0.74	1.41†
					(1.24–1.40)					(1.38–1.55)					(1.32–1.49)
Surgery	8544	0.53	5721	0.35	1.46†	25248	0.87	19484	0.67	1.30†	43220	1.30	30819	0.92	1.41†
					(1.34–1.60)					(1.21–1.39)					(1.33–1.50)
Psychiatry	8663	0.54	3061	0.19	2.72†	13866	0.48	6608	0.23	2.09†	12044	0.36	7799	0.23	1.56†
					(2.17–3.43)					(1.80–2.42)					(1.36–1.78)
ER	2821	0.17	1747	0.11	1.65†	4353	0.15	2900	0.10	1.52†	9287	0.28	6084	0.18	1.55†
					(1.47–1.84)					(1.38–1.68)					(1.41–1.69)
Other	61908	3.83	48439	2.99	1.27†	137488	4.75	105386	3.64	1.30†	214992	6.48	160348	4.79	1.35†
					(1.23–1.32)					(1.26–1.34)					(1.31–1.39)
**Total**	**266217**	**16.46**	**179376**	**11.07**	**1.47** †	**639552**	**22.11**	**471423**	**16.28**	**1.35** †	**1013701**	**30.56**	**819688**	**24.51**	**1.25** †
					**(1.43–1.51)**					**(1.33–1.38)**					**(1.23–1.27)**

* per one person-year. The total person-years for the LUTS vs. the non-LUTS group were: age<40 group 16172.9 vs. 16206.5; 40≤age<60 group 28919.4 vs. 28950.3; age≥60 group 33166.8 vs. 33449.6.

** Adjusted IRR: incidence rate ratio, reference group: non-LUTS, adjusted by age, gender, income, area, HTN, DM, hyperlipidemia and CAD.

# only women were included in gynecology. GP: general practice; ENT: ear, nose and throat; ER: emergent room;

† p-value <0.0029 with Bonferroni correction.

As expected, the hospitalization rates were higher in gynecology (LUTS group 45.88 vs. non-LUTS group 28.74) (no. per 1000 person-year) and urology (26.55 vs. 4.83), with an adjusted IRR of 1.60 (95% CI = 1.35–1.90) and 5.50 (95% CI = 4.65–6.50), respectively. The total hospitalization rate (except for gynecology and urology) (no. per 1000 person-years) for the LUTS group and the non-LUTS group were 311.24 vs. 209.91, with an adjusted IRR of 1.48 (95% CI = 1.40–1.58). In addition to gynecology and urology, the adjusted IRRs were also significantly higher in other departments (e.g. internal medicine (1.55, 95% CI = 1.45 = 1.66), surgery (1.56, 95% CI = 1.42–1.72), orthopedics (1.34, 95% CI = 1.22–1.47), and neurology (1.40, 95% CI = 1.22–1.60)). All *p*-values were <0.001. The adjusted IRR in psychiatry was 1.12 (95% CI = 0.75–1.67), and the *p*-value was 0.586. Details are listed in Table 4.

**Table 4:** Incidence rate ratio of hospitalizations among LUTS and non-LUTS individuals.

	LUTS (N = 39901)		Non-LUTS (N = 39901)		Unadjusted		Adjusted	
Department	No.	IR*	No.	IR*	IRR** (95% CI)	P-value	IRR*** (95% CI)	P-value
Gynecology#	1470	45.88	924	28.74	1.60 (1.40–1.82)	<0.001	1.60 (1.35–1.90)	<0.001
Urology	2078	26.55	380	4.83	5.49 (4.61–6.54)	<0.001	5.50 (4.65–6.50)	<0.001
Internal Medicine	13752	175.72	8896	113.17	1.55 (1.45–1.67)	<0.001	1.55 (1.45–1.66)	<0.001
Surgery	3527	45.07	2263	28.79	1.57 (1.42–1.73)	<0.001	1.56 (1.42–1.72)	<0.001
Colorectal	579	7.40	291	3.70	2.00 (1.39–2.88)	<0.001	1.99 (1.44–2.74)	<0.001
Cardiovascular	263	3.36	225	2.86	1.17 (0.87–1.59)	0.302	1.18 (0.88–1.57)	0.270
Thoracic	132	1.69	113	1.44	1.17 (0.67–2.06)	0.578	1.15 (0.72–1.86)	0.555
Gastrointestinal	211	2.70	135	1.72	1.57 (1.07–2.31)	0.022	1.56 (1.09–2.23)	0.015
Other surgeries	2342	29.93	1499	19.07	1.57 (1.41–1.74)	<0.001	1.57(1.42–1.74)	<0.001
Psychiatry	1377	17.60	1230	15.65	1.12 (0.70–1.79)	0.623	1.12 (0.75–1.67)	0.586
Orthopedics	1819	23.24	1364	17.35	1.34 (1.23–1.46)	<0.001	1.34 (1.22–1.47)	<0.001
Neurology	1153	14.73	827	10.52	1.40 (1.20–1.64)	<0.001	1.40 (1.22–1.60)	<0.001
Neurosurgery	732	9.35	551	7.01	1.33 (1.07–1.66)	0.010	1.34 (1.09–1.63)	0.050
Other	1997	25.52	1369	17.42	1.47 (1.30–1.65)	<0.001	1.47 (1.31–1.65)	<0.001
**Total**	**24357**	311.24	**16500**	209.91	**1.48 (1.40–1.57)**	**<0.001**	**1.48 (1.40–1.58)**	**<0.001**

* per 1000 person–year; the total person-years were: 78259.13 in the LUTS group, 78606.48 in the non-LUTS group;

** IRR: incidence rate ratio of the LUTS group vs. the non-LUTS group;

*** adjusted by age, gender, income, area, HTN, DM, hyperlipidemia, and CAD.

# only women were included in gynecology.

The rate of hospitalization increased with the advancement of age in both the LUTS and non-LUTS groups in urology, internal medicine, surgery, orthopedics, neurology, and neurosurgery; however, it decreased in gynecology and psychiatry. The adjusted IRRs were 1.77 (95% CI = 1.43–2.21) in the younger group, followed by the older group (1.47, 95% CI = 1.39–1.56), and the middle-aged group (1.44, 95% CI = 1.25–1.66) (Table 5). As age advanced, the IRRs for hospitalization decreased in internal medicine and neurology, but increased in gynecology. With the inclusion of LUT-related healthcare services, hospitalization visit rates among men and women increased in urology, but decreased in gynecology with the advancement of age in both LUTS and non-LUTS groups, although both adjusted IRRs increased. The adjusted IRRs were 1.44, 3.47, and 4.46 in gynecology; and 6.47, 6.69, and 13.1 in urology for the 3 age groups, a pattern that is similar to the one for outpatient visits (data not shown).

**Table 5:** Incidence rate ratio of hospitalizations among LUTS and non-LUTS individuals based on age group.

Grouping	Age<40					40≤Age<60						Age≥60						
	LUTS(N = 8099)		non-LUTS(N = 8108)		Adjusted IRR**(95%CI)	LUTS(N = 14552)		non-LUTS(N = 14538)			Adjusted IRR**(95%CI)	LUTS(N = 17250)		non-LUTS(N = 17255)		AdjustedIRR**(95%CI)		
Department	Visits	IR*	Visits	IR*		Visits	IR*	Visits		IR*		Visits	IR*	Visits	IR*			
Gynecology#	907	83.37	687	62.96	1.33†	400	31.13	178	13.88		2.29†	163	19.62	59	7.01	2.80†		
					(1.18–1.49)						(1.61–3.25)					(1.26–6.24)		
Urology	173	10.70	32	1.97	5.41†	669	23.13	146	5.04		4.61†	1236	37.27	202	6.04		6.16†	
					(3.52–8.32)						(3.51–6.04)						(4.79–7.92)	
Internal medicine	514	31.78	289	17.83	1.78†	2657	91.88	1504	51.95		1.77†	10581	319.02	7103	212.35		1.50†	
					(1.38–2.31)						(1.52–2.07)						(1.39–1.61)	
Surgery	354	21.89	182	11.23	1.90†	1003	34.68	679	23.45		1.48†	2170	65.43	1402	41.91		1.56†	
					(1.44–2.51)						(1.25–1.75)						(1.37–1.77)	
Psychiatry	462	28.57	186	11.48	2.46* ^1^	653	22.58	863	29.81		0.76	262	7.90	181	5.41		1.48	
					(1.12–5.41)						(0.42–1.36)						(0.80–2.72)	
Orthopedics	154	9.52	128	7.90	1.19	527	18.22	357	12.33		1.48†	1138	34.31	879	26.28		1.31†	
					(0.86–1.66)						(1.25–1.75)						(1.17–1.46)	
Neurology	40	2.47	14	0.86	2.77†	200	6.92	140	4.84		1.42* ^2^	913	27.53	673	20.12		1.37†	
					(1.43–5.37)						(1.05–1.93)						(1.15–1.63)	
Neurosurgery	55	3.40	31	1.91	1.71* ^3^	184	6.36	167	5.77		1.11	493	14.86	353	10.55		1.41* ^4^	
					(1.02–2.87)						(0.78–1.58)						(1.06–1.88)	
Other	239	14.78	181	11.17	1.29* ^5^	607	20.99	360	12.44		1.69†	1151	34.70	828	24.75		1.40†	
					(1.03–1.63)						(1.35–2.13)						(1.19–1.65)	
**Total**	**1818**	112.41	**1011**	62.38	**1.77** †	**5831**	201.63	**4070**	140.59		**1.44** †	**16708**	503.76	**11419**	341.38		**1.47** †	
					**(1.43–2.21)**						**(1.25–1.66)**						**(1.39–1.56)**	

* per 1000 person-year. The total person-years for the LUTS vs. the non-LUTS group were: Age<40 group 16172.91 vs. 16206.52; 40≤Age<60 group 28919.41 vs. 28950.34; Age≥60 group, 33166.81 vs. 33449.63;

** adjusted IRR: incidence rate ratio, reference group: non-LUTS, adjusted by age, gender, income, area, HTN, DM, hyperlipidemia and CAD.

# only women were included in gynecology.

† *p*-value <0.0029 with Bonferroni correction.

* *p*-values were 0.0249, 0.0240, 0.0169, 0.0407, and 0.0299 for *^1, 2, 3, 4, 5^, respectively.

## Discussion

This is the first large-scale nationwide study to estimate the risk of subsequent health-care services in terms of outpatient visits and hospitalizations in a nationwide population of medical attendance for LUTS, as compared with those without LUTS. The results show a significantly higher number of outpatient visits and hospitalizations among people with LUTS, with various effects brought on by the advancement of age, compared to non-LUTS controls. This is an observational study to offer prima facie evidence for correlation; however, the causality has not yet been confirmed. This study is in concordance with the 2006 U.S. National Health and Wellness Survey report on the effect of LUTS, including OAB/UI, on health outcomes [11]. The presence of LUTS, including OAB/UI, correlated significantly with increased resource use (e.g., emergency room visits (odds ratio 1.57, 95% CI = 1.47–.68) and medical provision visits (1.52, 95% CI = 1.41–1.63)) [11]. The increased risk of subsequent outpatient visits and hospitalizations highlights the significance of understanding LUTS under a broader perspective that includes multiple and overlapping systems, rather than considering them exclusively under the traditional concepts of bladder and urethra disorders [19].

People with LUTS tend to have more outpatient visits and hospitalizations in the LUTS-related specialties (e.g., gynecology and urology, which include transurethral resection of prostate (TURP)-related- lower urinary tract surgery in men and surgery for stress urinary incontinence and pelvic organ prolapse (POP) for women). Nevertheless, subsequent outpatient visit and hospitalization risks were still high after LUTS-related healthcare services were excluded. Additionally, LUTS predispose individuals to increased outpatient visits and hospitalizations in all other medical specialties. This extends the traditional concept that LUTS are confined to bladder and urethra disorders only. The underlying pathophysiology of LUTS may involve a complicated interaction among muscles, nerves, receptors, transmitters, and the brain [20]. Therefore, this study further supports the concept that LUTS are a non-organ-specific group of symptoms, which may impact the way approaches to LUTS reflect our recognition of the lower urinary tract as an integrated functional unit [20].

Results from this study also indicate that the rate and IRR of hospitalization differ in individual specialties with the advancement of age. The IRR of outpatient visits and hospitalizations increased with the advancement of age only in gynecology and urology, especially after taking into account LUTS-related healthcare services. The incidence rate of outpatient visits and hospitalizations in urology increased among men and women, but outpatient visits and hospitalizations in gynecology decreased among women with the advancement of age in both the LUTS and non-LUTS groups. These results are significant and require further investigation. It is possible that young and middle-aged women have obstetric and gynecological disorders more frequently than elderly women. Women in their 40s and 50s with LUTS and urogynecological disorders require hospitalization, however, the rate of hospitalization decreases for women over 60 years of age. The IRRs for outpatient visits and hospitalizations in gynecology increase with the advancement of age. On the contrary, both the incidence rate and IRR of urological outpatient visits and hospitalization increased from the 40–59 year-old age group to the ≥60 age group. BOO/BPH cause a higher rate of outpatient visits and hospitalization in urology with the advancement of age from the 40–59 year-old group to the ≥60 group. Men under 40 years of age without LUTS are typically healthy, and the rate of hospitalization is much lower than that of men of the same age with LUTS, resulting in a relatively higher IRR in this age group, compared to the other age groups. Younger people are usually not bothered by lower urinary tract dysfunction, and hospitalization in their case can be attributed to other diseases.

There are several explanations for the higher risk of outpatient visits and hospitalization. First, the incidence of comorbidities is higher among individuals with LUTS. LUTS share a number of risk factors with cardiovascular diseases [10]. Ng et al. reported that individuals with moderate-to-severe IPSS (≥8) have a statistically higher chance of having at least one cardiovascular risk factor during assessment (*p* = 0.001). Cardiovascular risk factors were prevalent in individuals with LUTS. A large number of these risk factors had remained unrecognized prior to urological consultation [21]. The role of vascular risk factors in LUTS is becoming increasingly recognized, and LUTS have been linked to obesity, HTN, hyperlipidemia, DM, and nicotine use [22], [23]. Ponholzer et al. also reported that people with an increased IPSS may have more vascular risk factors [24]. In a health screening project, the IPSS was identical in those with one or no vascular risk factors, however, it increased significantly in those with two or more risk factors among both men (*p* = 0.01) and women (*p = *0.05). The IPSS was identical between men with no vascular risk factors (6.2±4.1) and one risk factor (6.2±4.4); however, it increased to 7.7±5.5 (+24.2%) among those with two or more risk factors (*p* = 0.01). The IPSS increased from 4.8±4.6 among women with no vascular risk factor to 5.7±5.3 (+18.7%) among those with one risk factor and 7.0±5.7 (+45.8%) among those with two or more risk factors (*p* = 0.05). These data suggest that vascular risk factors play a role in the development of LUTS in both sexes [24]. The increase in the IPSS for people with two or more vascular risk factors, compared to those with none, was higher for women (+46%) than for men (+24%). LUTS affect men and women differently. Women with two or more vascular risk factors were, regarding the IPSS, twenty years older than those without risk factors, and the corresponding age difference among men was ten years [22]. Coyne et al. reported in an EPIC study that asthma was a predictor of bothersome OAB, whereas neurological conditions, recurrent urinary tract infections, and uterine prolapse were predictors for the seeking of treatment [25].

LUTS may be a syndrome of systemic disorders, rather than simply symptoms of a disease of the urinary bladder or urethra. For example, metabolic syndrome components worsen LUTS in women with type 2 DM [26]. LUTS and OAB had a higher prevalence among women in the metabolic syndrome group, and significantly higher storage and total American Urological Association Symptom Index (AUA-SI) scores were also noted for women in this group. Moreover, the number of metabolic syndrome components correlated strongly with the severity of LUTS. Similar results were found between metabolic syndrome and OAB. Metabolic syndrome may influence LUTS and OAB particularly in diabetic women, likely by compounding the effect of peripheral neuropathy [13]. In recent studies, we found serum C-reactive protein to be elevated in both men and women with LUTS and OAB, suggesting the possible presence of a systemic inflammatory process [27]–[29]. Serum nerve growth factor has also been found to increase in OAB patients who were refractory to medical treatment [30]. These evidence further show that LUTS and OAB are not simply a disease of the urinary bladder or urethra; instead, any systemic disorder can result in increased circulating inflammatory proteins and symptoms.

Second, LUTS may be a precursor condition, predisposing the development of certain medical and surgical conditions. Hu and Wagner reported LUTS/OAB to be associated with higher health risks (e.g., urinary tract infections, falls and fall-related injuries, including broken bones) [15]. Karatas et al. noted a higher prevalence of cardiovascular disease in patients with LUTS than in the general population in old age. Nocturia-induced sleep disturbances cause repeated waking and voiding attacks, non-dipping blood pressure variations and, consequently, increased sympathetic activity [31]. These studies suggest that vascular risk factors are associated with the presence and severity of LUTS. Wehrberger et al. reported a higher risk of cardiovascular disease and stroke events (adjusted hazard ratio 3.82, *p* = 0.01) for men with severe LUTS during a mean follow-up period of 6.1 years, after adjusting for age, diabetes, total- and low-density lipoprotein cholesterol in a 10-year follow-up longitudinal analysis, although moderate LUTS do not seem to be a risk factor for cardiovascular disease and stroke (adjusted hazard ratio 0.63, *p* = 0.16) [10]. It has also been shown that urinary incontinence is a predictor of an increased risk of mortality and poor functional recovery as well as of post-stroke institutionalization [32]. LUTS was considered a prognostic factor after acute first-ever stroke. Patients who regain normal bladder control in the first week have a prognosis that is comparable to that of patients who do not have micturition disturbances following a stroke [32]. Therefore, the increased economic burden raises the possibility that treating LUTS/OAB at an early stage can both improve patient care and minimize overall use of health-care resources [30].

Third, poorer QoL among people with LUTS can lower the threshold of outpatient visits and hospitalization requests. People with LUTS experience uncomfortable symptoms, poorer HRQL, and decreased work productivity [14], [33], [34]. These symptoms compromise HRQL and can cause considerable distress [12], [35]. The National Overactive Bladder Evaluation (NOBLE) Program found people with OAB with and without urge incontinence to have a poorer HRQL through clinically and significantly lower clinical SF-36 QoL scores [12]. Coyne et al. reported that men and women with bothersome OAB were significantly more likely to seek treatment (i.e., number of healthcare visits, urinary symptoms-related healthcare visits, treatment for urinary symptoms) [25]. In a 2006 report from the U.S. National Health and Wellness Survey, LUTS were found to be associated with an 8.03% overall work productivity loss, 12.88% activity impairment, and a decreased HRQL [11], [20]. Girman et al. found disease-specific HRQL to worsen with age. Adjusting for age, most disease-specific HRQL measures were significantly lower with the increase of symptom severity, despite potential cross-cultural differences in disease prevalence, medication use, disease perceptions, and willingness to report symptoms or worse HRQL [36]. The unexpectedly high frequency of outpatient visits (24.53 and 18.71 for LUTS and non-LUTS groups, respectively) may be a result of unique health-care-seeking cultures, the accessibility of health-care facilities, and the NHI-covered low co-pay system.

This study provides evidence that shows that people under medical attendance for LUTS are likely to experience an increased number of outpatient visits and hospitalizations. Further studies to increase understanding of the underlying and overlapping pathophysiologic mechanisms, associated comorbidities, and potential risks of developing other health problems are required [20]. This study reflects the pathophysiologic conditions in the entire body (e.g., metabolic, hormonal, cardiac, and respiratory processes) about LUTS, and it can broaden a clinician’s approach to managing individuals with LUTS [20]. Furthermore, LUTS likely indicate the existence or risk of other health problems, such as vascular or neurologic conditions. The negative effect of LUTS is apparent across several domains of HRQL and within the overall perception of bladder problems, general health statuses, and mental health [33]. Furthermore, this study broadens our perspective from a local organocentric focus to a recognition of clinical, economic, and humanistic scenarios [20].

Some limitations resulting from the characteristics of the registry of NHIRD database are inherent in this study [18]. Firstly, a lack of coding or miscoding may exist due to the potential negligence of the clinician or medical staff. The lack of coding of some significant terms, (e.g., urgency) may exist. The term OAB was generally coded as hypertonicity of bladder (596.51); therefore, no OAB diagnosis can be made according to ICS terminology [37]. Secondly, other potential confounders such as education, marital status, alcohol use, tobacco use, and measures of baseline health status (e.g., obesity and parity) were not available in the dataset. These confounders could have an impact on the results [18]. Third, the criterion may be too loose because three outpatient visits with the coding of LUTS and no other specified diagnoses was used as the inclusive criterion. Nevertheless, the risks of outpatient visits and hospitalizations were still significant. This further strengthens the critical impact of LUTS on health care. Lastly, a proportion of male patients were diagnosed with LUTS/BPH, but no symptom subtypes were assigned to them by physicians.

### Conclusion

In this study, we found a significantly higher number of outpatient visits and hospitalizations among individuals with LUTS, as compared to non-LUTS controls. This study highlights the prevalence of LUTS in subsequent health-care services among different specialties and age groups. It also provides a broader understanding of LUTS within multiple and overlapping systems, as well as explanations for how these symptoms impact further health-care services, such as the existence of comorbidities, precursor conditions, and early symptoms of subsequent medical or surgical conditions.
